# Dynamic changes in the hormones of black-necked cranes during reproduction

**DOI:** 10.1093/conphys/coac040

**Published:** 2022-07-02

**Authors:** Yihua Wang, Guogang Zhang, Hongxing Jiang, Dongping Liu, Xingbo Hu, Fawen Qian

**Affiliations:** Key Laboratory of Forest Ecology and Environment of National Forestry and Grassland Administration, Ecology and Nature Conservation Institute, Chinese Academy of Forestry, No.2 Dongxiaofu, Haidian District, Beijing, 100091, China; Key Laboratory of Forest Ecology and Environment of National Forestry and Grassland Administration, Ecology and Nature Conservation Institute, Chinese Academy of Forestry, No.2 Dongxiaofu, Haidian District, Beijing, 100091, China; Key Laboratory of Forest Ecology and Environment of National Forestry and Grassland Administration, Ecology and Nature Conservation Institute, Chinese Academy of Forestry, No.2 Dongxiaofu, Haidian District, Beijing, 100091, China; Key Laboratory of Forest Ecology and Environment of National Forestry and Grassland Administration, Ecology and Nature Conservation Institute, Chinese Academy of Forestry, No.2 Dongxiaofu, Haidian District, Beijing, 100091, China; Beijing Shoufa Tianren Ecological Landscape Co., Ltd., Beijing, 102600, China; Key Laboratory of Forest Ecology and Environment of National Forestry and Grassland Administration, Ecology and Nature Conservation Institute, Chinese Academy of Forestry, No.2 Dongxiaofu, Haidian District, Beijing, 100091, China

**Keywords:** sex hormone, glucocorticoid metabolites, droppings, black-necked crane, Artificial breeding

## Abstract

Black-necked cranes (*Grus nigricollis*) are national first-level protected wild animals in China. Artificial breeding has been adopted by many zoos and reserves to achieve *ex-situ* conservation of black-necked cranes, but the breeding rate of the species in cages is low. This study used non-invasive methods combined with behavioural observations to investigate changes in sex hormones and glucocorticoid metabolites in the droppings of black-necked cranes during the breeding cycle, with the results showing that (i) levels of estradiol and testosterone in black-necked cranes increased significantly when they entered the breeding period, and these levels could be used as an important physiological indicator to effectively monitor the physiological status of females and males during the reproductive period, thus providing a theoretical basis for the timing of semen collection; (ii) the level of progesterone in the mid-reproduction stage was significantly higher than that in other stages in female black-necked cranes after successful mating, and this level could be an effective indicator of the mating status of female black-necked cranes; (iii) droppings’ glucocorticoid metabolites in the breeding period showed different dynamics between paired and singly caged black-necked cranes, indicating that the physiological phenomenon of reproduction could result in a certain amount of physiological burden on black-necked cranes. These results provide a theoretical basis for the selection of physiological parameters in the artificial breeding of black-necked cranes.

## Teaser

Changes in hormones of black-necked crane during different reproductive stages.

## Introduction

The black-necked crane has been identified as a globally vulnerable species (VU) by the International Union for Conservation of Nature and listed as a class I endangered and protected species in China. As the only plateau crane in the world (Choki *et al.,* 2011; Li, 2014; Song *et al.,* 2014; Zhang *et al.*, 2017), its survival status is a focus of conservation biology (Choki *et al.,* 2011; Li, 2014; Song *et al.,* 2014; Zhang *et al.*, 2017). Due to the harsh natural environment in which black-necked cranes live, and the slow growth of the population in the wild, many zoos and reserves have used cages to protect and breed them (Gan *et al*., 1983; Zhang, 2000a). However, due to the low reproduction rate of black-necked cranes (Gan *et al*., 1983, Gee, 1983), artificial insemination has become an auxiliary method to increase their reproduction rate (Zhang, 2000b; Zhang *et al.,* 2014). Current artificial insemination methods for black-necked cranes are based mainly on experience and have low success rates. In the process of artificial insemination, the timing of semen collection and insemination is very important, and properly timing these procedures could drastically improve the success rate of semen collection, as well as the success rate of egg fertilization (Gee and Temple, 1978). Artificial insemination is of vital importance to the proliferation of endangered birds (Zhang, 2000b). Steroid hormones are also transported to the liver to be metabolized and eventually excreted in the form of metabolites through the kidney into the urine or through the bile into the intestine and excreted in the faeces. In the case of birds, the excreta collected are a mixture of faeces and urine. Ludders *et al.* (2001) previously validated non-invasive research methods for sandhill cranes and showed that it is feasible. This non-invasive sampling study has solved many problems that cannot be solved due to the limitation of traditional sampling and has been widely used in various fields of ornithological research, including stress physiology (Liu *et al.,* 2018), metabolic physiology (Wasser *et al.,* 2010), genetic diversity (Bejapereira *et al.,* 2009), reproductive physiology (Krepschi *et al.,* 2013; Penfold *et al.,* 2013) and animal immunity studies (Liu *et al.,* 2018). In the present study, using non-invasive methods of stool research, the reproductive physiological characteristics of black-necked cranes were investigated during different reproductive stages to provide a theoretical basis for parameter measurements and procedure timing during the artificial breeding of black-necked cranes.

**Table 1:** Basic information for the black-necked cranes used in this study

Individual code^a^	Cage number	Gender	Age	Paired or not	Health status	Successfully lay eggs (yes/no)	Oviposition experience (yes/no)
Q11	3	♀	7	No	Good	No	Unkown
3	4	♀	≥20	No	Good	No	Unkown
Q10	5	♂	7	No	Good	—	—
296	1	♀	15	Yes	Good	No	No
Q0009	1	♂	7	Yes	Good	—	—
13F	2	♀	15	Yes	Good	Yes	Yes
13M	2	♂	14	Yes	Good	—	—

^a^Pedigree number of the black-necked crane (unique to each bird).

## Materials and methods

### Materials

The black-necked cranes used in this study were residents at the Ming Tombs breeding base of Beijing Zoo. Located next to the Changling Mausoleum of the Ming Tombs in Changping, Beijing, this breeding base is a quiet and secluded place for breeding, scientific research, animal quarantine and animal recuperation at Beijing Zoo. When this study was initiated, a total of eight black-necked cranes (four females and four males) were reared in this base, of which four were paired in 2017 (one pair were naturally paired successfully in 2016 and raised in cages in 2017, while the other pair were artificially raised in cages in 2017) and the remaining four (two females and two males) were individually reared. The cages for paired black-necked cranes are 20 m long, 5 m wide and 3.5 m high, while the cages for individual black-necked cranes are 12 m long, 3.1 m wide and 3.5 m high. The subjects of this experiment were all artificially raised; the feed used for them consisted mainly of dried corn pellets, steamed bread, farina for pheasants, beef scraps, lettuce, rape, green corn, frozen fish, frozen shrimp, boiled chicken eggs, living loach, etc. Since a single male crane died in mid-June 2017, we collected and analysed faeces samples from the remaining seven healthy black-necked cranes. The individuals sampled were all healthy during the sampling period. Basic information about the black-necked cranes used in this study is shown in Table 1.

### Collection and preservation of faecal samples

From early March to mid-August 2017, fresh faecal samples were collected every 3 days for each of the seven black-necked cranes in captivity. The sampling time was 8:00–10:00 am (Beijing time). An uncontaminated portion of each fresh faeces sample was placed in a 10-ml centrifuge tube with the sample number and date marked, after which it was immediately placed into a refrigerator at −20°C.

### Extraction and determination of faecal hormones

The method used here was an improved version of that described by Rettenbacher *et al.* (2004) and Ludders *et al.* (2001). In addition, the effectiveness of this method has been verified and applied in a variety of birds (Dehnhard *et al.,* 2003). First, 0.5 g wet faeces was mixed with 5 ml solvent (in which the volume of ethanol:distilled water was 9:1) in a 10-ml test tube, which was gently vortexed for ~20 minutes and centrifuged at 231 × g for 15 minutes. The supernatant was moved into an evaporating dish to dry. Next, 1 ml 0.02PBST + 5% methanol solution was added, and the sample was dissolved with shaking. Each aliquot was stored at −20°C.

Enzyme-linked immunosorbent assays (ELISA) were used to determine the content of glucocorticoid metabolites in the droppings in each aliquot, and faecal water content was determined at the same time. The final result was converted into the content of hormones per gramme of dry faeces. The enzyme-labelled analyser was a Labsystems Multiskan MS-352, the plate washer was a Thermo Labsystems-AC8 and the corticosterone kit was produced by Shanghai Bangyi Biotechnology Co., Ltd. The main technical parameters of the corticosterone kit were as follows: determination range, 20–640 μg/l; sensitivity, <1.0 μg/l; intra-assay coefficient of variation, <10%; and inter-assay coefficient of variation, <15%. With regard to specificity, there was no cross-reaction. ELISAs was used to determine the content of progesterone, estradiol and testosterone hormone metabolites in each aliquot. At the same time, the water content of the faeces was measured, and the final result was converted into the content of hormones per gramme of dry faeces. The enzyme-labelled analyser was a Thermo Fisher Multiskan FC, the plate washing machine was a Beijing Tianshi ZMX-988b and the kits for progesterone, estradiol and testosterone were produced by Beijing Northern Institute of Biotechnology. The technical parameters for progesterone determination were as follows: determination range, 0.5–30 ng/ml; sensitivity, ≤0.1 ng/ml; intra-assay coefficient of variation, <10%; and inter-assay coefficient of variation, <15%. With regard to specificity, there was no cross-reaction with other soluble structural analogues. The technical parameters of estradiol determination were as follows: determination range, 50–3000 pg/ml; sensitivity, ≤4 pg/ml; intra-assay coefficient of variation, <10%; and inter-assay coefficient of variation, <15%. With regard to specificity, there was no cross-reaction with other soluble structural analogues. The technical parameters for testosterone determination were as follows: determination range, 0.25–32 ng/ml; sensitivity, ≤0.125 ng/ml; intra-assay coefficient of variation, <10%; and inter-assay coefficient of variation, <15%. With regard to specificity, there was no cross-reaction with other soluble structural analogues.

### Method of behavioural observation

The behaviour of the target black-necked cranes was observed and recorded at the same time every day during the sampling period. The details of behavioural observation are as follows:

We observed the breeding behaviours of black-necked cranes during the early stage of the breeding, and the courtship, mating and nesting behaviours were recorded. The observation time was 9:30–10:30 am and 3:30–4:30 pm every day. The selection of behavioural observation time was based on Yang’s research on the breeding behaviour of black-necked cranes (Yang, 2005) and based on the experience of zoo keepers. In addition, we did not make a detailed and specific analysis of the frequency of specific behaviours, and only made a record of the behaviours with or without.

Behaviour definition:

- Courtship show-off behaviour: Dance behaviour of raising and lowering head for many times.
- Mating behaviour: The male spreads his wings and leaps onto the back of the female, flapping his wings to adjust his balance. After the male stands firm, the tails of the female and male meet

### Data analysis

Based on the faecal water content of the control groups, the data were converted into the sex hormone content (estradiol, progesterone, testosterone and glucocorticoid metabolites in the droppings) per gramme of dry faeces (μg/g, ng/g, ng/g and μg/g). SPSS software was used to perform one-way Analysis of Variance and multiple comparisons for the content of sex hormones in the faecal samples with the significance level set to *P* = 0.05. Levene’s homogeneity of variance test was used to determine the homogeneity of the sample mean square error. Dunnett’s T3 test was used for multiple comparisons of data from different sampling periods. The faecal sex hormone data showed a Gaussian distribution and were denoted as mean ± SE.

## Results

Based on the results of the measurement of gonadal steroid hormone metabolites and behavioural observation of caged black-necked cranes during the experimental sampling period, we divided the reproductive period (from March to August) of paired caged black-necked crane individuals into four different stages: non-breeding stage, early reproductive stage, mid-reproductive stage and late reproductive stage.

The reproductive behaviours of the black-necked cranes at different stages were as follows:

Non-breeding stage: The black-necked cranes paced back and forth in the cage, fed and sometimes chirped.

Early reproductive stage: The male cranes often showed more excited behaviours in comparison with those shown during the non-reproductive stage, such as increased call frequency, flapping their wings and dancing against their female cranes. The paired birds sometimes moved towards each other, and they also exhibited singing and singing in unison. The male and female black-necked cranes stretched their necks forward while chirping, they vocalized (‘coo…coo…’) at the same time and they approached each other slowly; the female cranes spread their wings and half-squatted with their tails facing the male crane, at which point the male crane half-spread his wings and jumped onto the back of the female crane, where he balanced with both feet for copulation. The paired black-necked cranes designated as 13F (♀) and 13 M (♂) were observed mating from mid-May to early June. The black-necked cranes designated as 296 (♀) and Q0009 (♂) were observed mating in mid-May. Individual black-necked cranes numbered Q11 and Q10 were kept in separate cages from mid-April to mid-May and were observed to show reproductive behaviour during this time period. No reproductive behaviour was observed in the black-necked crane individual designated as No. 3.

Mid-reproductive stage: The black-necked cranes designated as 13F (♀) and 13 M (♂) mated successfully and laid eggs. This pair of black-necked cranes laid five eggs in succession. The zoo artificially incubated the eggs, so this pair of black-necked cranes did not incubate their eggs during this period. Another pair of black-necked cranes, designated as 296 (♀) and Q0009 (♂), did not lay eggs, although mating behaviour was observed.

Late reproductive stage: Since the eggs were hatched artificially, the black-necked cranes got no chance to raise their young cranes during this period.

The specific time allocation for the different breeding stages was as follows Table 2:

**Table 2:** Specific time allocation for the different breeding stages

No.	Non-breeding stage	Early reproductive stage	Mid-reproductive stage	Late reproductive stage
13F(♀)	Early Mar–15 Apr	16 Apr–3 Jun	4–27 Jun	28 Jun and after
13M (♂)	Early Mar–15 Apr	16 Apr–13 Jun	14–30 Jun	1 Jul and after
296 (♀)	Early Mar–20 Apr	21 Apr–31 May	1 Jun–9 Jul	10 Jul and after
Q0009 (♂)	Early Mar–10 Apr	11 Apr–14 May	15 May–Jul	10 Jul and after
Q11 (♀)	Early Mar–10 Apr	11 Apr–31 May	1–30 Jun	1 Jul and after
3 (♀)	Early Mar–10 Apr	11 Apr–31 May	1–30 Jun	1 Jul and after
Q10 (♂)	Early Mar–10 Apr	11 Apr–31 May	1–30 Jun	1 Jul and after

### Sex hormone dynamics in black-necked crane individuals

#### Estradiol level dynamics in female black-necked cranes

**Figure 1:**
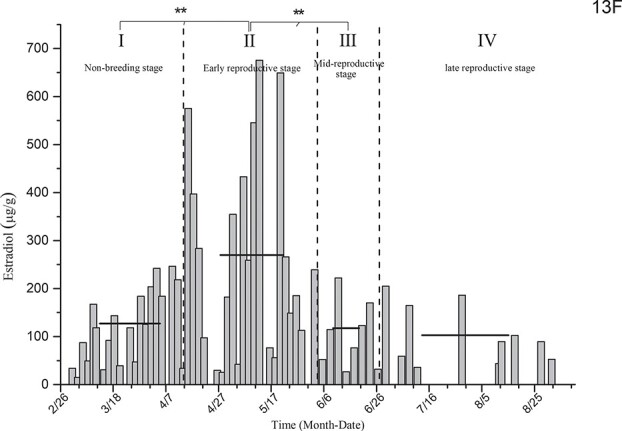
Comparison of average estradiol levels in the non-breeding stage and three other breeding stages for individual 13F

Estradiol level determination for the black-necked crane designated as 13F (Fig. 1) showed that the average levels of estradiol in the non-breeding stage (I), early reproductive stage (II), mid-reproductive stage (III) and late reproductive stage (IV) were 127.15 ± 16.89 μg/g, 269.83 ± 47.67 μg/g, 117.46 ± 26.36 μg/g and 102.96 ± 19.42 μg/g, respectively. The estradiol level in the early reproductive stage was significantly increased and was significantly higher than that in the other stages: *P*(II & I) = 0.002, *P*(II & III) = 0.008 and *P*(II & IV) = 0.003.

**Figure 2:**
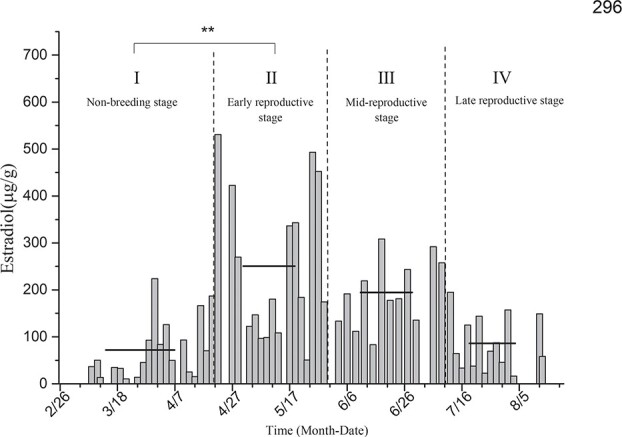
Comparison of average estradiol levels in the non-breeding stage and three other breeding periods for individual 296

Estradiol level determination for the black-necked crane individual designated as 296 (Fig. 2) showed that the average levels of estradiol in the non-breeding stage (I), early reproductive stage (II), mid-breeding stage (III) and late reproductive stage (IV) were 72.12 ± 14.41 μg/g, 250.68 ± 39.44 μg/g, 194.55 ± 20.65 μg/g and 86.17 ± 15.34 μg/g, respectively. The level of estradiol increased significantly after subject 296 entered the reproductive period, and the difference between the early reproductive stage and the non-breeding stage was extremely significant (*P* < 0.01). The average estradiol level in the mid-breeding stage was significantly higher than that in the non-breeding stage, while there was no significant difference between the average estradiol levels of the late reproductive stage and non-breeding stage (*P* = 0.684).

**Figure 3:**
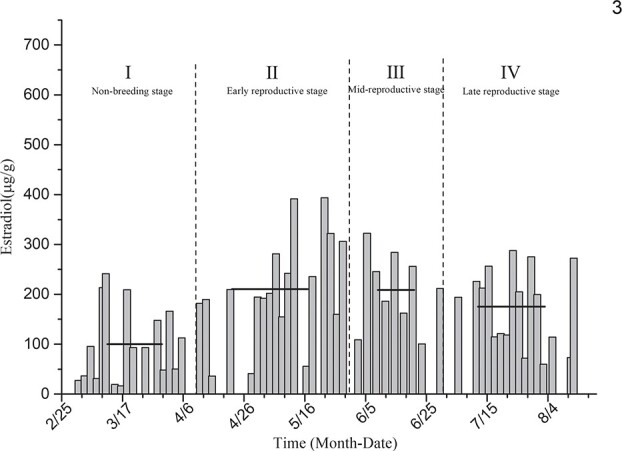
Comparison of average estradiol levels in the non-breeding stage and three other breeding periods for individual No. 3

Estradiol level determination for the singly reared black-necked crane individual designated as No. 3 (Fig. 3) showed that the average levels of estradiol in the non-breeding stage (I), early reproductive stage (II), mid-breeding stage (III) and late-reproductive stage (IV) were 100.11 ± 18.76 μg/g, 210.55 ± 24.61 μg/g, 208.65 ± 25.44 μg/g and 175.12 ± 19.61 μg/g, respectively. The level of estradiol in this individual was higher in the early and middle stages of reproduction, during which it was significantly different from that measured during the non-breeding stage (*P* < 0.01).

**Figure 4:**
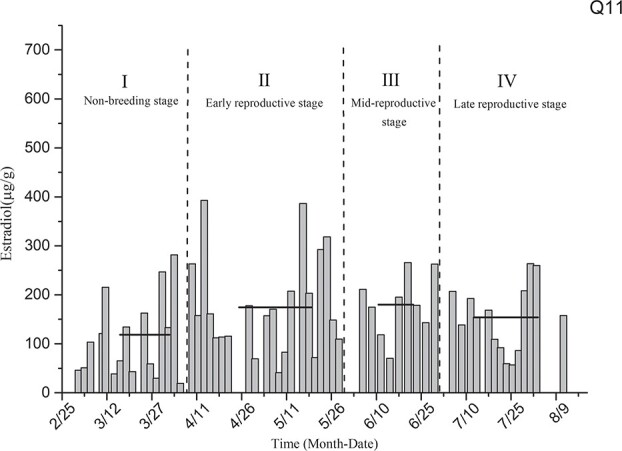
Comparison of average estradiol levels in the non-breeding stage and three other breeding periods for individual Q11

Estradiol level determination for the singly reared black-necked crane individual designated as Q11 (Fig. 4) showed that the average levels of estradiol in the non-breeding stage (I), early reproductive stage (II), mid-breeding stage (III) and late-reproductive stage (IV) were 118.44 ± 21.17 μg/g, 174.50 ± 22.57 μg/g, 180.06 ± 21.28 μg/g and 153.87 ± 18.10 μg/g, respectively. The levels of estradiol in this individual in the reproductive and non-breeding stages did not differ significantly (*P* > 0.05).

#### Progesterone level dynamics in female black-necked cranes

**Figure 5:**
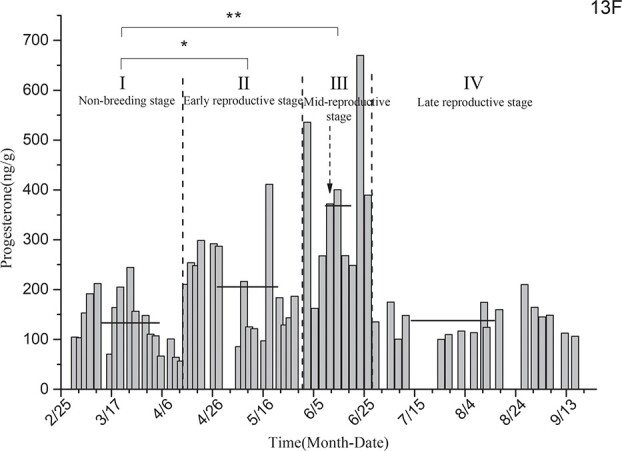
Comparison of average progesterone levels in the non-breeding stage and three other breeding periods for individual 13F

Progesterone level determination for the black-necked crane individual designated as 13F (Fig. 5) showed that the average levels of progesterone in the non-breeding stage (I), early reproductive stage (II), mid-breeding stage (III) and late reproductive stage (IV) were 132.89 ± 13.85 ng/g, 205.58 ± 22.32 ng/g, 368.19 ± 52.36 ng/g, and 137.81 ± 7.63 ng/g, respectively. This individual had the highest progesterone level in the mid-breeding stage, and her progesterone level was increased significantly in the early reproductive stage in comparison with that measured during the non-breeding stage. The average level of progesterone in the non-breeding stage was significantly different from that measured during the early reproductive stage (*P* = 0.016) and extremely different from that measured during the mid-breeding stage (*P* < 0.01), but showed no significant difference from that measuring during the late reproductive stage (*P* = 0.865).

**Figure 6:**
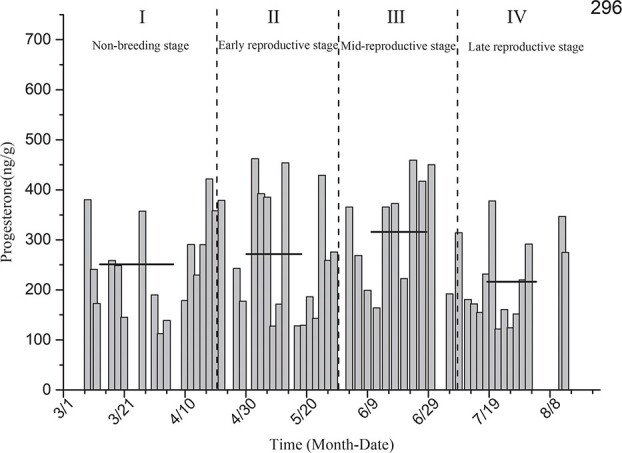
Comparison of average progesterone levels in the non-breeding stage and three other breeding periods for individual 296

**Figure 7:**
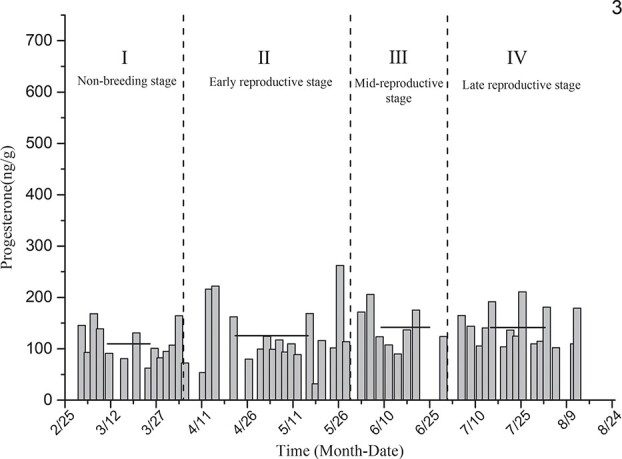
Comparison of average progesterone levels in the non-breeding stage and three other breeding periods for individual No. 3

**Figure 8:**
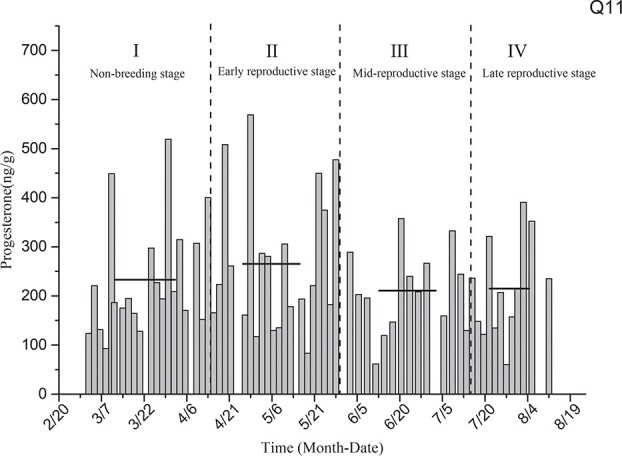
Comparison of average progesterone levels in the non-breeding stage and three other breeding periods for individual Q11

Progesterone level determination for the black-necked crane individual designated as 296 (Fig. 6) showed that the average levels of estradiol in the non-breeding stage (I), early reproductive stage (II), mid-breeding stage (III) and late reproductive stage (IV) were 250.82 ± 23.31 ng/g, 271.42 ± 31.55 ng/g, 315.92 ± 30.16 ng/g and 216.07 ± 23.22 ng/g, respectively. No significant differences in the level of progesterone among different reproductive periods were identified for this individual (*P* > 0.05).

Progesterone level determination for the black-necked crane individual designated as No. 3 (Fig. 7) showed that the average levels of estradiol in the non-breeding stage (I), early reproductive stage (II), mid-breeding stage (III) and late reproductive stage (IV) were 109.59 ± 9.11 ng/g, 125.56 ± 14.04 ng/g, 141.76 ± 13.79 ng/g and 141.25 ± 9.30 ng/g, respectively. The levels of progesterone in the reproductive period and non-breeding stage did not differ significantly, and there was no significant difference among the progesterone levels measured during the different stages of the reproductive period.

Progesterone level determination for the black-necked crane individual designated as Q11 (Fig. 8) showed that the average levels of estradiol in the non-breeding stage (I), early reproductive stage (II), mid-breeding stage (III) and late-reproductive stage (IV) were 232.88 ± 25.66 ng/g, 265.16 ± 31.58 ng/g, 211.01 ± 22.46 ng/g and 215.05 ± 28.70 ng/g, respectively. The levels of progesterone in the reproductive period and non-breeding stage did not differ significantly.

#### Testosterone level dynamics in male black-necked cranes

**Figure 9:**
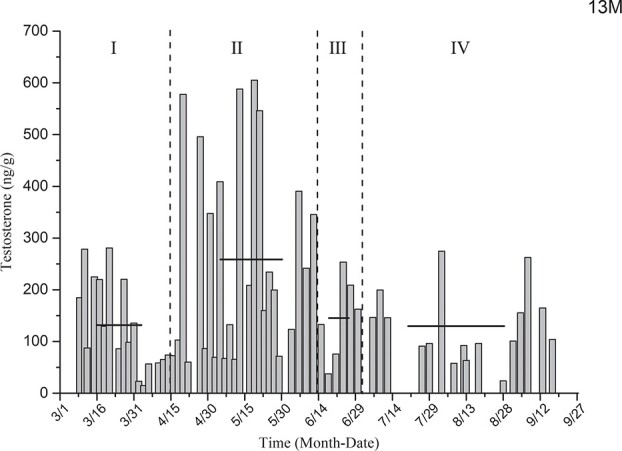
Comparison of average testosterone levels in the non-breeding stage and three other breeding periods for individual 13 M

Testosterone level determination for the black-necked crane individual designated as 13 M (Fig. 9) showed that the average levels of testosterone in the non-breeding stage (I), early reproductive stage (II), mid-breeding stage (III) and late reproductive stage (IV) were 131.59 ± 21.01 ng/g, 258.36 ± 39.20 ng/g, 145.18 ± 32.93 ng/g and 129.65 ± 17.46 ng/g, respectively. The level of testosterone was lower in the non-breeding stage and significantly increased in the early reproductive stage. The level of testosterone in the non-breeding stage and that in the early reproductive stage differed significantly (*P* < 0.01), but there was no significant difference between the testosterone levels during the non-breeding stage and mid−/late-reproductive stage (*P* = 0.833, *P* = 0.967).

**Figure 10:**
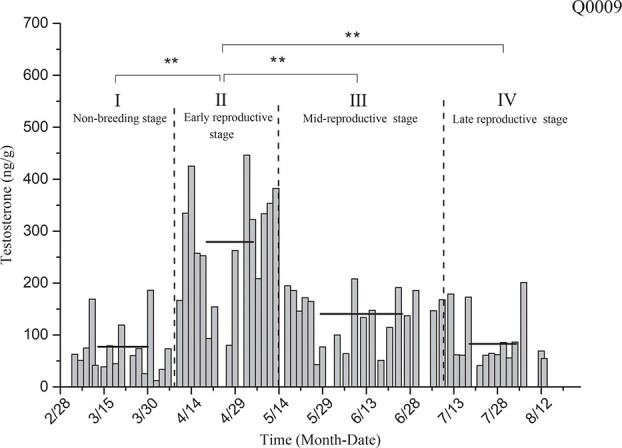
Comparison of average testosterone levels in the non-breeding stage and three other breeding periods for individual Q0009

Testosterone level determination for the black-necked crane individual designated as Q0009 (Fig. 10) showed that the average levels of testosterone in the non-breeding stage (I), early reproductive stage (II), mid-breeding stage (III) and late reproductive stage (IV) were 77.22 ± 12.71 ng/g, 278.97 ± 30.52 ng/g, 140.48 ± 11.22 ng/g and 82.76 ± 13.33 ng/g, respectively. The testosterone level of this individual in the early reproductive stage was significantly higher than that in the non-breeding stage and other stages of the reproductive period (*P* < 0.01).

**Figure 11:**
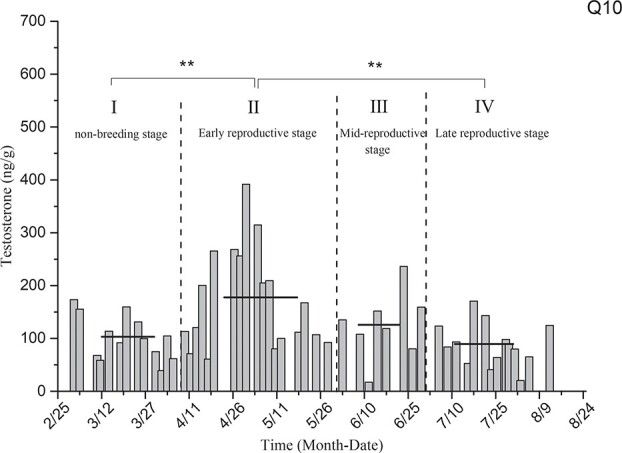
Comparison of average testosterone levels in the non-breeding stage and three other breeding periods for individual Q10

Testosterone level determination for the black-necked crane individual designated as Q10 (Fig. 11) showed that the average levels of testosterone in the non-breeding stage (I), early reproductive stage (II), mid-breeding stage (III) and late reproductive stage (IV) were 103.24 ± 10.94 ng/g, 177.75 ± 23.44 ng/g, 125.85 ± 22.44 ng/g and 89.38 ± 10.93 ng/g, respectively. The average level of testosterone in the early reproductive period was generally higher than that measured during the other stages, and it was significantly different from that in the non-breeding stage (*P* = 0.003) and late reproductive stage (*P* = 0.001).

### Glucocorticoid metabolites in the droppings dynamics in black-necked cranes during breeding and non-breeding stages

#### Glucocorticoid metabolites in the droppings in caged pair-reared female black-necked cranes during breeding and non-breeding stages

**Figure 12:**
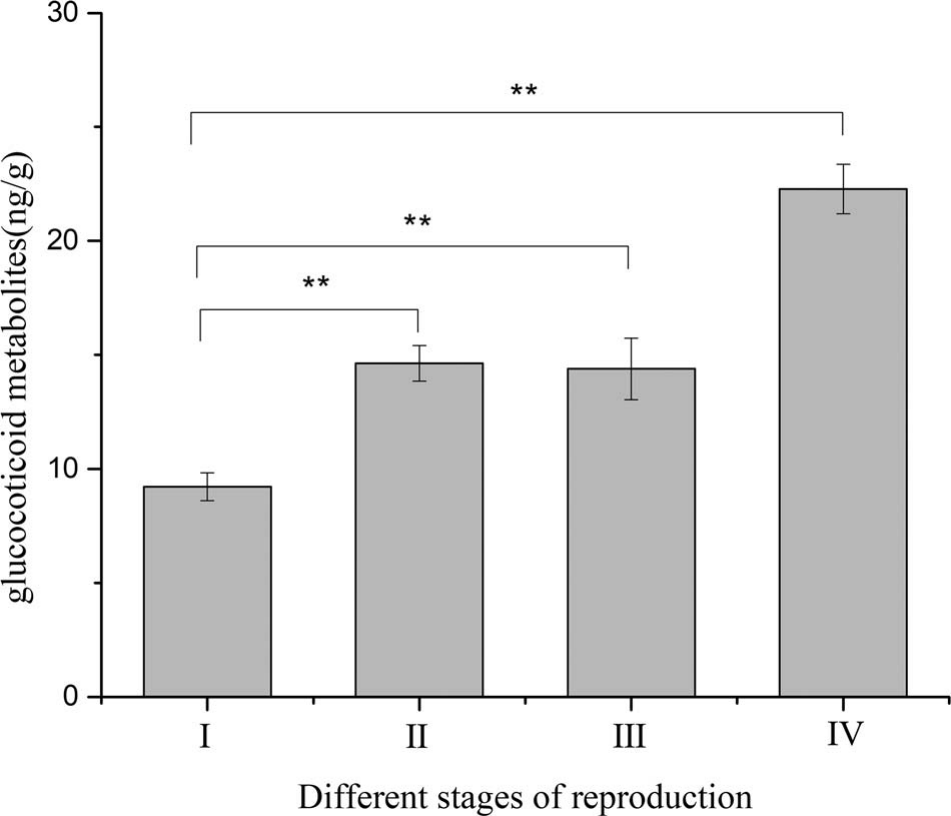
Comparison of average glucocorticoid metabolites levels during different stages of the breeding period for pair-reared female cranes

Faecal glucocorticoid metabolites level determination for the two pair-reared female black-necked cranes (Fig. 12) showed that the average levels of glucocorticoid metabolites in the non-breeding stage (I), early reproductive stage (II), mid-breeding stage (III) and late reproductive stage (IV) were 9.23 ± 0.61 μg/g, 14.62 ± 0.78 μg/g, 14.38 ± 1.34 μg/g and 22.27 ± 1.09 μg/g, respectively. The average level of glucocorticoid metabolites in the reproductive period was higher than that in the non-breeding stages, and the average level in the late reproductive stage was significantly higher than that in the other stages of the breeding period.

#### Glucocorticoid metabolites dynamics of ovipositing and non-ovipositing individuals in paired cage rearing

**Figure 13:**
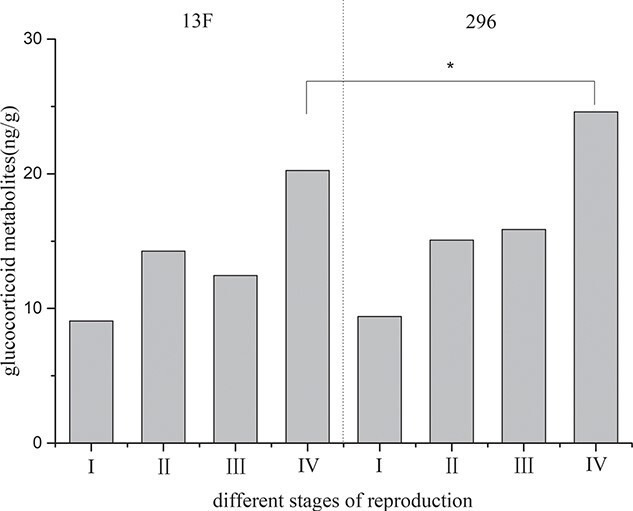
Comparison of average glucocorticoid metabolites levels between 13F and 296 individual

The results (Fig. 13) showed that the glucocorticoid metabolite content in non-oviposition female (269) (24.59 ± 1.54 ng/g) was significantly higher than that in oviposition female (13F) (20.24 ± 1.38 ng/g) (*P* = 0.016). And there was no significant difference in other reproductive and non-reproductive stages.

#### Glucocorticoid metabolites dynamics in caged pair-reared male black-necked cranes during breeding and non-breeding stages

**Figure 14:**
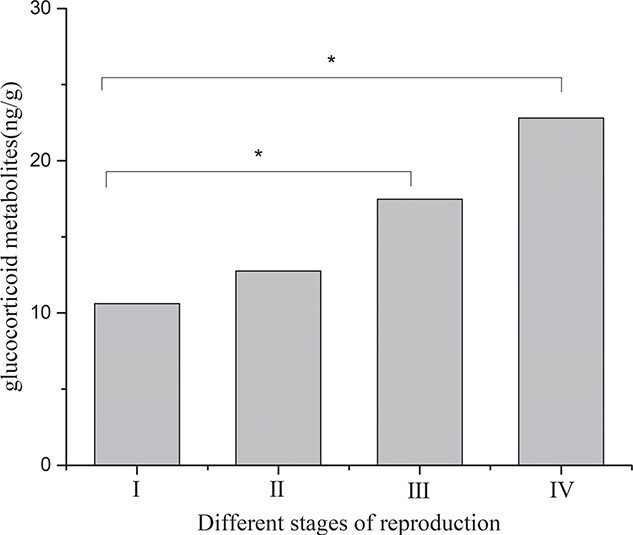
Comparison of average glucocorticoid metabolites levels during different stages of the breeding period for pair-reared male cranes

Faecal glucocorticoid metabolites level determination for the two pair-reared male black-necked cranes (Fig. 14) showed that the average levels of glucocorticoid metabolites in the non-breeding stage (I), early reproductive stage (II), mid-breeding stage (III) and late reproductive stage (IV) were 10.62 ± 0.87 μg/g, 12.75 ± 0.73 μg/g, 17.48 ± 1.17 μg/g and 22.80 ± 1.05 μg/g, respectively, indicating that the average level of glucocorticoid metabolites during the reproductive period was significantly higher than that in the non-breeding stages.

#### Glucocorticoid metabolites dynamics in singly caged female black-necked cranes during breeding and non-breeding stages

**Figure 15:**
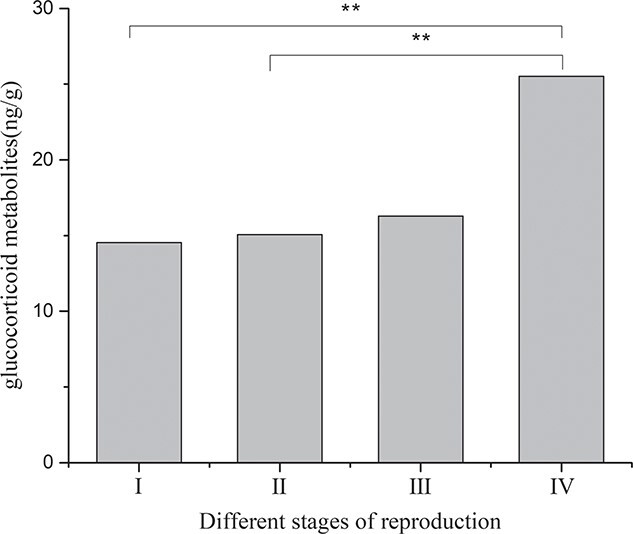
Comparison of average glucocorticoid metabolites levels during different stages of the breeding period for singly reared female cranes

Faecal glucocorticoid metabolites level determination for the two singly reared female black-necked cranes (Fig. 15) showed that the average levels of glucocorticoid metabolites in the non-breeding stage (I), early reproductive stage (II), mid-breeding stage (III) and late reproductive stage (IV) were 14.53 ± 0.72 μg/g, 15.07 ± 0.64 μg/g, 16.29 ± 1.20 μg/g and 25.51 ± 1.02 μg/g, respectively, indicating that the level of glucocorticoid metabolites presented an upward trend after the subjects entered the reproductive period, while the levels of glucocorticoid metabolites in the early reproductive and mid-breeding stages were not significantly different from that in the non-breeding stage and the level of glucocorticoid metabolites increased significantly in the late stage of reproduction.

#### Glucocorticoid metabolites dynamics in singly caged male black-necked cranes during breeding and non-breeding stages

**Figure 16:**
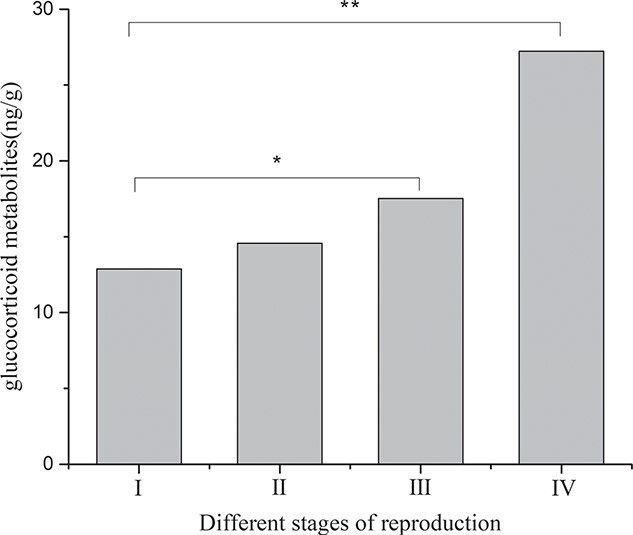
Comparison of average glucocorticoid metabolites levels during different stages of the breeding period for singly reared male cranes

Faecal glucocorticoid metabolites level determination for the two singly reared male black-necked cranes (Fig. 16) showed that the average levels of glucocorticoid metabolites in the non-breeding stage (I), early reproductive stage (II), mid-breeding stage (III) and late reproductive stage (IV) were 12.87 ± 1.05 μg/g, 14.57 ± 0.82 μg/g, 17.52 ± 1.73 μg/g and 27.23 ± 1.72 μg/g, respectively, indicating that the levels of glucocorticoid metabolites in the early and middle stages of reproduction were elevated and the glucocorticoid metabolites level peaked in the late stage of reproduction, during which it was significantly different from that measured during the other stages.

## Discussion

### Characteristics of sex hormone dynamics in black-necked cranes

Methods for the detection of sex steroid hormones in animal faeces have been successfully established and widely used in captive and free-living wild animals, domestic animals and laboratory animals, including mammals (Emery and Whitten, 2003; Yarnell *et al.,* 2016), birds (Hirschenhauser *et al.,* 1999; Zhang and Ding, 2012; Yamak, 2015; Knutie and Gotanda, 2018) and amphibians (Kummrow *et al.,* 2015; Szymanski *et al.,* 2006).

The reproductive behaviour of birds is determined by many factors, including external environmental factors (such as light duration, season, food and temperature, etc.) (Breuner *et al.,* 2013) and internal physiological factors (Hurlbert, 2016), but external ecological factors require internal physiological mechanisms to mediate their effects. Neuroendocrine activity is the internal basis for bird reproduction. Related studies have shown that the reproductive behaviours of animals are regulated by estradiol and progesterone (Penfold *et al*., 2013; Brown, 2013; Brown *et al.,* 2016), and estradiol regulates the initiation of estrus in females of many different species (Walker *et al.,* 2002). In the research of canaries, author measured faecal estradiol-17b and progesterone in laying canaries to better understand how onset of incubation might regulate clutch size, results suggest that factors initiating incubation also cause the decline in E production by small follicles, which in turn may inhibit yolk sequestration in large follicles (Sockman and Schwabl, 1999). Hirschenhauser *et al.* (1999) measured and analysed steroid hormones in grey goose faeces during different seasons, and found that steroid hormones showed differentiated characteristics in different social classes and different reproductive states. In this study, we found that the estradiol and progesterone levels of paired female black-necked cranes increased after they entered the breeding period and the average level of estradiol in the early reproductive stage was significantly higher than that in the non-breeding stage. Although the level of progesterone increased during the early reproductive stage, there was no significant difference compared with the average level in the non-breeding stage, indicating that estradiol should serve as a better monitor for the reproductive physiological status of black-necked crane females, which could provide a theoretical basis for the selection of appropriate artificial insemination times in artificial breeding, and thus may effectively increase the fertilization rate of this species in captivity. In addition, we speculate that the increase in the progesterone level during the early reproductive stage might act synergistically with estradiol to induce the performance of estrus and courtship behaviour (Xu, 2014).

In this study, monitoring the estradiol levels of black-necked cranes in the context of successful and unsuccessful mating during different reproductive stages showed that female black-necked cranes that successfully bred and mated had higher estradiol levels in the early reproductive stage, but the estradiol level in the middle reproductive stage quickly returned to the average level of the non-breeding stage. In female black-necked cranes that did not succeed in breeding and mating, the estradiol level increased in the early reproductive stage and remained high in the mid-reproductive stage, after which it dropped back to the average level of the non-breeding stage in the late reproductive stage. This phenomenon might be related to unsuccessful mating and failing to lay eggs, but further study will be required to understand this effect. Through our analysis of faecal estradiol levels and behavioural observations during the breeding period, we found that the estradiol levels of singly reared female black-necked cranes with estrous behaviour increased in the early reproductive stage and were significantly higher than the levels measured during the non-breeding stage. However, the estradiol levels of the female black-necked cranes without reproductive behaviour did not differ during the reproductive period and non-breeding stage. This result indicates that estradiol could be a good indicator of the reproductive physiological state of female black-necked crane individuals, and the same phenomenon has been reported in other research (Christensen *et al*., 2012; Goutte *et al.,* 2010).

Comparison of the progesterone levels of females that were successful and unsuccessful at mating indicated that the level of progesterone in the females that successfully mated remained relatively high for a long time after successful mating and were significantly different from that in the non-breeding stage. Nevertheless, mating was not successful (no eggs laid) when the level of progesterone in the black-necked crane females did not change significantly during the reproductive period compared with that in the non-breeding stage, although mating behaviour was observed. In addition, the progesterone levels of singly reared female black-necked cranes showed no significant difference between the non-breeding stage and the reproductive period, as well as among different stages of the reproductive period, which indicated that progesterone can be used as a potential indicator to monitor whether female black-necked cranes can successfully lay eggs.

The success of animal mating is closely related to the quality of individual male sperm for mammals (Asa *et al.,* 2010) and birds (Brown *et al.,* 2015). Relevant studies have shown that the quality of whooping crane sperm is related to the season or time of reproduction (Brown *et al.,* 2015), rather than age or inbreeding coefficient. Testosterone is a good indicator of the reproductive physiological status of male animals, and testosterone content is closely related to the quality of sperm (Anderson and Navara, 2011). The results of this study showed that the testosterone levels in male black-necked cranes increased significantly after they entered the breeding period. This phenomenon has also been observed in birds such as red-crowned cranes (Wang *et al.,* 2014) and yellow-bellied pheasants (Zhang, 2001). Non-invasive monitoring provides the basis for determining the optimal semen collection time for artificial breeding of black-necked cranes. The testosterone level of singly reared male black-necked cranes also increased significantly in the early stage of reproduction, during which reproductive behaviour was observed, indicating that testosterone was positively correlated with reproductive behaviour. Therefore, the testosterone level of black-necked cranes could be used to monitor the reproductive physiological status of male individuals.

### The dynamics of glucocorticoid metabolites in black-necked cranes

Glucocorticoids are secreted by the adrenal cortex and reflect the stress level of an animal, which is determined by the external environment and internal physiology. In birds, glucocorticoids are excreted mainly by faeces in the form of metabolites (Xu, 2014). In this study, we found that the average level of glucocorticoid metabolites during the breeding period of black-necked cranes paired in cages was significantly higher than the average level during the non-breeding stage. In contrast, the level of glucocorticoid metabolites in the breeding period of singly reared black-necked cranes was significantly increased, but was not significantly different from that measured during the non-breeding stage. This phenomenon may be related to the greater physiological stress experienced by the paired individuals in comparison with the singly caged individuals. In addition, we also found that the average level of glucocorticoid metabolites in the black-necked cranes during the breeding period was quite different from that measured during the other stages. We speculate that this phenomenon due to cage-rearing might be an external environmental factor that irritates black-necked cranes, leading to a significant increase in glucocorticoid metabolites. What is more, we found that the glucocorticoid metabolites in females with unsuccessful oviposition were higher than those in females with successful oviposition, suggesting that reproductive failure may further enhance the stress of black-necked cranes; specific reasons for these phenomena merit further study.

## Data Availability

The data underlying this article will be shared on reasonable request to the corresponding author.
